# Antimicrobial stewardship in the community setting: a qualitative exploratory study

**DOI:** 10.1186/s13756-025-01524-7

**Published:** 2025-02-11

**Authors:** Rose I Okonkwo, Henry Ndukwe, Gary Grant, Sohil Khan

**Affiliations:** 1 School of Pharmacy and Medical Sciences, Griffith University, Gold Coast, QLD Australia; 2 Manipal College of Pharmaceutical Sciences and Prasanna School of Public Health, Manipal Academy of Higher Education, Manipal, India

**Keywords:** Antimicrobial stewardship, Antimicrobial resistance, Antibiotic stewardship, Antimicrobial prescribing, Community setting, Community AMS, Health system

## Abstract

**Background:**

Existing evidence underscores inappropriate antimicrobial prescribing and use in the community setting. Increased and inappropriate antimicrobial use are major factors contributing to the emergence and transmission of antimicrobial resistance (AMR). Antimicrobial stewardship (AMS) programmes are critical for mitigating AMR, enhancing patient outcomes, and reducing healthcare costs. Despite the existing Australian National Action Plan on AMR, optimisation of antimicrobial use in the community setting remains inadequately investigated. This study explored health professionals’ perspectives on community AMS practices and systems, identifying challenges and areas for improvement.

**Methods:**

This qualitative study utilised semi-structured interviews to explore the perspectives of 17 different health professionals from diverse community practice settings in South-East Queensland, Australia. Interviews were audio-recorded, anonymised, and transcribed verbatim. Data were thematically analysed, with NVivo 12 utilised for organisation and analysis. Data were then mapped and examined using the Elements of Medicines Stewardship (EMS), which aligns with United States Centers for Disease Control and Prevention– Core Elements of Antibiotic Stewardship. This was reported following the consolidated criteria for reporting qualitative research checklist.

**Results:**

Four main themes described the health professionals’ insights on community AMS practices and systems. Thematic analysis from these findings reveals a state of ambiguity and fragmentation in the community AMS practices and systems. In comparison to the hospital-based AMS system, the Australian community AMS system appears to be in its nascent stages of development. Applying the EMS is essential for developing and implementing community AMS strategies to enhance practices and systems.

**Conclusion:**

The study identified key health system factors that impact the implementation of community AMS programmes and highlighted the need for developing community-specific governance and frameworks that integrate multidisciplinary strategies to support effective implementation and enhance patient outcomes. This research will inform community AMS intervention strategies, influencing policy and practice to advance sustainable healthcare and address antimicrobial resistance.

**Supplementary Information:**

The online version contains supplementary material available at 10.1186/s13756-025-01524-7.

## Background

The World Health Organisation (WHO) has declared that antimicrobial resistance (AMR) is one of the top global public health threats facing humanity [1]. Globally, antimicrobial resistant infections caused 1.27 million deaths in 2019 [2]. AMR is projected to result in 10 million deaths per year by 2050, costing the global economy a cumulative US $100 trillion [2, 3]. In Australia, it is estimated that AMR will cause over 10,400 deaths between 2015 and 2050, and at the same time healthcare costs predicted to be around US $370 million [4]. Optimising the use of antimicrobial medicines, one of the five strategic objectives of the WHO Global Action Plan on AMR, is an essential approach for preventing and minimising AMR [5, 6]. AMS programmes can have a significant impact on reducing the incidence of AMR, improving patient outcomes and reducing healthcare costs. They are recognised as a key strategy for combating the global threat of antimicrobial resistance.

Antimicrobial consumption has been associated with AMR, and data shows that high rates of antimicrobial use are often associated with high rates of inappropriate use [7, 8]. Increased and inappropriate antimicrobial use are major factors contributing to the emergence and transmission of AMR [1]. Overuse and inappropriate use of antimicrobials remain evident, and there is an indication of inappropriate antimicrobial prescribing and use in the community setting. Evidence shows that in the primary care setting, many common conditions are still being treated inappropriately, and in residential aged care or nursing homes, levels of inappropriate antimicrobial use remain high [9–16]. In comparison to most European nations, the United Kingdom and Canada; Australia had a greater community usage of antibiotics in 2021 with 32.9% of its population receiving at least one antimicrobial dispensed in the community [8, 9]. This has increased to over one-third (36.4%) of the Australian population in 2023 [17]. Also, antimicrobial prescribing in Australia remains high compared with European countries [9, 17]. Compared to the hospital, most antimicrobial use occurs in the community, and notably over 80% of antibiotic prescriptions occur in Australia’s community setting [7].

AMR is a growing problem globally. While AMR in the hospital setting has been extensively studied, data on AMR in the community setting are limited [7, 8]. The community setting is pivotal to AMS efforts because it represents a large portion of antimicrobial use. Effective AMS in this setting is crucial to mitigating AMR and ensuring the long-term efficacy of antimicrobial therapies. Consequently, it is imperative to prevent the development and spread of antimicrobial-resistant organisms, which depends on the capacity to recognise and minimise inappropriate antimicrobial prescribing and use. Optimising medicines use is a priority for enhancing quality of care and health outcomes, while ensuring value and sustainability, as it is the most conventional healthcare intervention worldwide [18]. Despite the existing Australian National Action Plan on AMR, optimisation of antimicrobial use has not been adequately understood or explored in the community setting. Therefore, given the high burden of AMR and the significant antimicrobial use at community level, an improved understanding of AMS practices and systems in this setting is essential. The role of health professionals in mitigating AMR and enhancing patient outcomes within the community is critical and cannot be overstated. There is a limited understanding of the perspectives of health professionals on community AMS practices and systems as it is less studied. This study aimed to explore the current practices, perceptions, and views of health professionals in the community setting regarding community AMS practices and systems, and to highlight challenges, areas for improvement and strategies to optimise AMS.

## Methods

### Study design, setting, and participants

This qualitative study using semi-structured interviews was conducted in South-East Queensland (SEQ), Australia which has an estimated population of approximately 3.9 million people [19]. The choice of SEQ was based on relevance to the research question, feasibility, and practical considerations, ensuring the study can achieve its objectives effectively. In Australia, a significant portion of the experience in AMS has been in the hospital sector where different health professionals contribute to AMS. For this reason, we considered the selection of different health professionals in the community setting that can influence community AMS. Target participants were general practitioners, pharmacists, nurses, AMS and infection prevention control coordinators, laboratory scientists/ managers who were involved in providing AMS in their organisation, supporting or working in the community setting in SEQ, Australia (for example general practice clinics, outpatient clinics, aged care homes and community pharmacies). Furthermore, engaging different health professionals and diverse community practice settings facilitated the inclusion of varied perspectives and contextual variations, thereby enhancing credibility and improving validity. The study used a purposive sampling approach to recruit participants [20]. In addition, to attain adequate sampling, convenience sampling and snowball techniques were utilised to identify participants considering the practice setting of health professionals, time and resource constraints [21, 22]. Initial contact of the participants was made with the aid of an advertisement flyer shared to the relevant health facilities (general practice clinics, primary health networks, outpatient clinics, community pharmacies, aged care homes, nursing homes etc.), through the respective health professional societies/ associations/ organisations (e.g., Pharmaceutical Society of Australia, The Royal Australian College of General Practitioners Pharmaceutical Society of Australia etc.), and the State Government Health Department. Flyers were sent by email and/or placed on a notice board in these health facilities calling for participants to contact the research team to express an interest in the research. Interested participants contacted the research team through email with the details on the flyer. Participants were formally recruited by R.I.O. by contacting them through email using a short letter of introduction noting the importance of the individual as a key stakeholder and citing the participant’s information sheet, obtaining consent prior to participation and plans for appointment. This advance letter paved the way for subsequent phone calls to arrange the interview meeting.

### Data collection

The semi-structured interviews followed interview guides that explored participants’ views on current AMS practices, challenges in its implementation and strategies to optimise AMS practices and systems in the community setting. First, an unstructured interview was done with an accredited consultant pharmacist with discussions probing for the subjects and concepts of AMS in the community setting in SEQ; this aided the design of the interview guide. The interview guide was developed by R.I.O., and interview questions were broadly informed by identified gaps from the literature review. This guide was adapted to suite the different health professions and specific interview guides for different health professions were generated. The interview guides were subjected to both content and face validity assessments and subsequently standardised, incorporating the feedback received. This process was carried out iteratively through evaluations by other members of the research team– S.K., G.G. and H.N. In addition, this was evaluated through a pilot interview with another pharmacist. This was explored to refine the questions, assess feasibility and ensure the subject being investigated was adequately captured by the proposed interview guides, and recommended changes were incorporated. The detailed interview guides are provided in the supplementary file. We used pharmacists for the preliminary exploratory interviews because of their integral role in AMS regardless of the practice setting [23–25]. The two pharmacists engaged for the exploratory interviews were identified through convenience sampling approach using contacts known to the research team and these interviews were not included in the main study. For the main study, a total of 17 health professionals were interviewed virtually between 30 September 2022 and 23 March 2024 and took approximately 25–60 min. Participants availability and inclusion of diverse perspectives i.e. a wider range of participants and contexts, impacted the data collection period. Before each interview, the participant information sheet, participant consent document and study advertisement flyer were sent to interested participants by email. The one-to-one interviews were conducted by R.I.O. through Microsoft Teams based on participants’ preference at a date and time convenient for participants. Consent to participate was verbally obtained before the commencement of the interview. All interviews were audio-recorded, anonymised, and transcribed by R.I.O.; and preliminary familiarisation with data begun during the transcription process. Participants were interviewed until thematic saturation was reached; this was determined using a simple method developed by Guest and colleagues [26]. The required sample size for data saturation was determined based on evidence and practical considerations, including time constraints and limited access to the study population, with saturation achieved when additional interviews yielded little or no new relevant information related to the study objectives [27, 28]. Demographic information, such as sex, age, and years of work experience, were not collected to prioritise participants’ comfort to ensure open and honest discussions. This approach allowed participants to focus solely on sharing their experiences and perspectives relevant to the study, focusing on the research themes and highlighting practice settings within the community. Memoing and reflexive practice were adopted when the study was conceptualised and utilised throughout data collection, analysis, and interpretation to ensure transparency, credibility and rigor [29, 30]. The quality of the transcripts was checked by R.I.O., who read and reread each transcript while listening to the corresponding audio recordings. Member checking or respondent validation of the interview transcripts was conducted to explore the credibility of data, this was achieved by sending the interview transcripts to participants to check for accuracy and resonance with their experiences [31]. Four of the 17 participants validated their transcripts, and their feedback was consistent with the interview data. Participants were compensated for their time as per ethics guidance.

### Data analysis

The qualitative analysis utilised inductive approach to explore and understand current AMS practices and systems in the community setting in South-East Queensland, highlighting challenges, areas for improvement and strategies to optimise AMS. Each transcript was read multiple times by R.I.O. to gain a deeper understanding and analyse participants’ responses as it recognised the common patterns across the stakeholders [32]. Interview data was inductively analysed in an open and descriptive method looking for patterns relative to the research questions which allowed for an objective exploration of participants’ perceptions and views [32–35]. This was achieved by coding the messages provided by the participants and then clustering them to produce sub-themes, and through the data interpretation process, generated themes. Also, meetings among the research team were held to deliberate and agree on the coding framework and organising into key themes. Consensus was reached among the research team through iterative discussions and checks, which continued throughout data analyses and reporting of this study, enhancing the transparency and reliability of the analysis process. Any disagreements between the researchers (R.I.O. and H.N.) were resolved through discussions with a third member of the research team (S.K.). The computer-aided qualitative data analysis software– NVivo 12 (QSR International Pty Ltd., 2018) facilitated the organising, managing and analysis of data [36]. Moreover, data were later mapped and examined using the Elements of Medicines Stewardship (EMS), which aligns with the United States Centers for Disease Control and Prevention (CDC)– Core Elements of Antibiotic Stewardship [37, 38]. The elements of medicines stewardship programmes include multidisciplinary leadership teams, stakeholder engagement, tailored communication strategies, proven methodologies in behavioural change and implementation science, and ongoing monitoring, evaluation and reporting [37]. The elements are used to address particular challenges within any specific therapeutic area to help to ensure appropriate and efficient use of medicines. The explanation of each construct, underlying principles and practical implications have been discussed [37]. This study is reported in accordance with the consolidated criteria for reporting qualitative research (COREQ) checklist [39]. Ethical approval for this study was obtained from the Institutional Human Research Ethics Committee (reference number: 2022/537).

## Results

A total of 17 health professionals were interviewed and the sample reflected diverse practice settings in the community. This encompassed 5 doctors (general practice setting), 6 pharmacists (1 in aged care, 1 in general practice and 4 in community pharmacy settings), 4 nurses (3 in aged care and 1 in general practice settings) and 2 laboratory scientists/ managers supporting community-based pathology services.

Data analysis revealed four main themes which described insights of health professionals in the community setting regarding community AMS practices and systems. Participants’ quotes were referenced using codes showing the participant’s health profession (doctor = D, nurse = N, pharmacist = P and laboratory scientist/ manager = L), the participant’s number which indicated the order in which they were interviewed and their practice area within the community setting (general practice = GP, community pharmacy = CP, aged care = AC, residential medication management review = RMMR, home medicines review = HMR and pathology services = PS). A summary of the themes, sub-themes and sample quotes is depicted in Table 1.

**Table 1:** Summary of themes, sub-themes and sample quotes from participants

Themes	Sub-themes	Sample quotes
Stakeholder mapping and governance structures	Community AMS stakeholder mapping	*External high-level AMS team*: • “I would still suggest that there should be sort of like a regular stakeholder meeting that would involve people in the infectious diseases, microbiologists, laboratory pathologists… And there should then be feedback to the rest of the stakeholders, it would help our prescribing pattern” (D,3,GP). • “I believe there is so much more the government could do to support us in many ways, and I’m not talking about financial support. I’m talking about, to have a go-to-person from the public health units or the department of health units. So, when people have questions or when people need further education and resources, we can go to them.” (N,15,AC).
		*Multidisciplinary AMS team*: • “I think it needs to be obviously a teamwork and get more education on it and more designated staff…” (P,1,AC, RMMR, HMR). • “I think it’s feasible, but I don’t think it would be specific to AMS because of the size of general practices.…Whether or not the pharmacist-nurse-general practitioner team could work on AMS, absolutely! I think it would just come as part of a package of other things.” (P,12,GP, HMR)
	Community AMS governance structures	*AMS as a health facility requirement*: • I hope that these discussions of AMS become a little bit more structured in the very near future as in a more legislative requirement of the aged care standards.” (N,15,AC) • “Feels like the only way that it’s going to happen is actually gonna be if it’s legislated. People don’t do work unless they have to do work.” (L,10,PS).
		*Roles and responsibilities of stakeholders*: • “…we don’t have a designated person for infection prevention control,…all of us are responsible for infection prevention and control.” (N,6,AC). • “Yeah, I think we can do better than what we are doing now. There is no real coordination even though the therapeutic guideline is supposed to help guide our prescribing pattern. But you still have different prescribers prescribing different things.…there is really no sort of coordination. And I think it’s because of lack of regular education around this topic for prescribers to know that we have to be careful about prescribing pattern and our prescribing pattern can actually increase antibiotic resistance in the community.” (D,3,GP).
Communication strategy and system	Communication among stakeholders	*Communication among health providers*: • “[It is] not very effective, no. So, usually we only call the doctor if there is a problem, but they don’t like hearing from us, to be honest.…I don’t think that engagement is very good. It’s probably better in a collaborative setting such as a hospital.” (P,11,CP). • “I guess engagement with general practitioners is always a big challenge… We find a lot of them feel that if a resident or a client asks for antimicrobials that they need to prescribe them.” (N,14,AC).
		*Communication with patients*: • “In community pharmacy, I think the communication with consumers or clients can still be effective… Dedicated time I think is the key which you don’t always have in a community pharmacy with the general practitioner or the consumer. Yeah, workload time pressures for all parties– consumers, general practitioners and pharmacists.” (P,2,CP, HMR). • “……sometimes it could be challenging explaining to patient that there is no clear indication for them to receive antibiotic at this moment.” (D,17,GP).
	Community AMS communication system	*Communication strategy*: • “I can only go to the doctor and talk about these things. There is no platform to provide feedback per say.” (N,5,GP). • “Communication pathways can be anything from a corridor chat to a formal internal messaging system which can be linked to patients’ charts.” (P,12,GP, HMR).
		*External high-level communication strategy*: • “…being more open to collaboration and networking with all the sites. I think that would be the greatest improvement that we could do is having more open collaboration between multidisciplinary, all the different strings that are involved in AMS.” (L,10,PS). • “I guess a database. A database that should be able to be accessible from clinicians, private pathology companies. Perhaps a database like that, that’s accessible for all of those bodies that do that work.” (L,9,PS).
Prescribing, dispensing and surveillance systems	Audit and surveillance data and system	*Tracking and reporting of data*: • “On our own we don’t track the prescription data or the antimicrobial resistance patterns…… So yeah, we don’t track such.… then encouraging every facility to be able to track their antimicrobial use and resistance patterns.…So, having the authorities find a way to kind of include that in maybe the appraisals or something so that practices can do that.” (D,4,GP). • “…I think at the moment [currently] the audit that the aged care is collecting, the data is very basic.…Not enough information to review the appropriate use of antibiotics” (P,1,AC, RMMR, HMR)
		*Local community AMR data or pattern*: • “If I had knowledge of the local antimicrobial resistance pattern, then I wouldn’t be starting off with basic 101 antibiotic if it was already resistant to it. Because I have no knowledge of what the antimicrobial resistant pattern is in my area.…I just go blindly and hope the first course works. If it doesn’t, then I’ll try another one and then I am also adding to the resistance!” (D,13,GP). • “I think it would really help us. I’m not aware of the resistant pattern in our community. In saying that, if we have this information clearly available, it will guide us as to which antibiotics would give in particular cases. You know, we won’t be giving patients antibiotics that we know people are commonly resistant to in the community. We can go for the antibiotics that will treat their infection. So, we need this information to be freely made available.” (D,3, GP).
	Prescribing and dispensing strategies	*Prescribing*,* medication reviews and dispensing practices*: • “The fact that you don’t give antimicrobials somebody else would give. Difficult patients and poor education,…I think sometimes the pressure that patients put on doctors can sometimes make doctors succumb to pressure.” (D,16,GP). • “…. patient-doctor shopping like for example, somebody received antibiotics about 2 weeks ago for a particular illness, but because he or she has not been improving or getting well might go to another doctor for antibiotics. So, sometimes it takes time to cross check through my health record to confirm this patient have received antibiotics two weeks ago. It adds to the consultation time in order to cross check their previous medication history.” (D,17,GP).
		*Prescribing and dispensing software*: • “There is more than one software used for dispensing.…. we have clashing software now, so that’s why there need to be some linkages. Because there’s nothing linked and it’s a waste of data that nothing is linked. It’s a huge job, the government needs to support that software development and the linking.” (P,2,CP, HMR). • “I can search for what prescriptions have been created by the GPs, but I can’t search for pathology results. I’d love to be able to do more searches that involve pathology results…. I’ve been told it’s not possible from an IT perspective, but that’s just in the software that GP practices use.…. I’m told that when the program conducts the search, it can’t read pathology results within the patient’s electronic medical record.” (P,12,GP, HMR).
Resources	Resource challenges	*Awareness and education for stakeholders*: • “Well, educating the prescribers in the sense that making them aware of what tools, like you heard me say I am not aware of some of these tools and I’m sure many of my colleagues will say the same.” (D,4,GP). • “I think our challenge is twofold. It is educating patients, which is easy, but educating prescribers, which is a little bit harder to do.” (P,11,CP).
		*Uptake of existing resources*: • “The company that I work for has done quite a lot of resources for antimicrobial stewardship since the clinical care for AMS has come through in aged care… So, we’ve given them to the aged care, but it has been not a great uptake of it. It seems like just from looking back at how they were using antimicrobials despite the resources that we have provided the aged care.” (P,1,AC, RMMR, HMR). • “…they [the government] should publicise it first. I’m not sure many doctors are aware of this. So, my thoughts should be first they should try to make sure every doctor gets to be aware that such kind of documents or measures are out there.…The point is, if you don’t know about it, you don’t really have an opinion on it. So, I don’t even know that such things exist, and they should do more to publicise it.” (D,4,GP).
	Resource gaps	*Human resources*: • “…there is often short staff in the aged care. Also with the aged care doctors, a lot of them are quite elderly. And I feel like they may not be up to date with the latest guideline on the treatments. Yeah, I think that’s also a huge factor.” (P,1,AC, RMMR, HMR). • “……the government needs to roll out so many adverts around there, employing more healthcare workers that can promote the use of antimicrobial resources that people can read whether online or through brochure or whether through healthcare magazines that are out there, for people to actually have a lot of support.” (N,6,AC).
		*Non-human resources*: • “We have the TG [therapeutic guideline or national guideline] at the moment, which is very good. But I would also prefer if there is more like a statewide [local] guideline that we can have easy access to and also statewide [local] resistance patterns. Yeah. If we have, like a policy statement or a policy guideline that we can easily default to, I think it would really help us.” (D,3,GP). • “I think even just getting access to things like therapeutic guidelines would be really helpful.…it would be really helpful to have more resources available to the nurses and the general practitioners on appropriateness of antimicrobial prescribing and reviewing pathology. (N,14,AC).”

### Stakeholder mapping and governance structures

A significant challenge identified in community AMS was the lack of community AMS stakeholder mapping and governance structures. Stakeholder mapping involves identifying and prioritising stakeholders to assess their influence on the programme and optimise communication and engagement strategies. Conversely, governance structures encompass the system of rules, processes, roles, and responsibilities that guide decision-making within a programme.

#### Community AMS stakeholder mapping

Some participants expressed the need for an influential external AMS team to provide leadership, oversight and support for AMS activities within the community. This team would provide education, expert guidance, and centralised support, enabling healthcare workers to implement AMS strategies more effectively and improve outcomes. Also, participants highlighted the absence of a coordinated team of health professionals managing AMS at the health service delivery level. Most interviewees acknowledged the importance of teamwork, consisting of various roles–doctors, pharmacists, and nurses–working collaboratively to optimise antimicrobial use and improve health outcomes.

#### Community AMS governance structures

Participants emphasised the non-existence of formal, mandatory frameworks that direct how AMS should be implemented in the community setting, unlike in hospitals where AMS is a standard requirement. This gap creates inconsistency in monitoring and managing AMS practices across varied health facility settings in the community. In addition, they expressed concerns over the lack of clearly defined roles and coordination among healthcare professionals and other stakeholders involved in AMS efforts within the community setting. This confusion results in variable practices and underscores lack of accountability, poor performance and outcomes in community AMS efforts.

### Communication strategy and system

One challenge in implementing community AMS practices is the lack of an adequate communication strategy and system. Effective communication is crucial for coordinating efforts among healthcare professionals, sharing information, and ensuring consistent adherence to AMS guidelines. The absence of a robust communication strategy and system hinders the efficient dissemination of critical data on antimicrobial prescribing, AMR trends, and AMS feedback, leading to inconsistent practices, missed opportunities for quality improvement, and misalignment among healthcare professionals with AMS objectives.

#### Communication among stakeholders

A significant barrier to effective AMS practices is the poor communication among healthcare providers, particularly doctors, pharmacists, and nurses. Communication among health providers is often informal and unstructured, typically occurring only when there is a concern. All participant community pharmacists reported ineffective communication with doctors, which worsens with after-hours or instant script doctors, especially on weekends, hindering timely AMS interventions. Doctors, while recognising pharmacists’ accessibility, emphasised the need for a structured communication system. In aged care, communication gaps are wider; nurses and pharmacists reported minimal interaction with doctors, who rarely attend Medicine Advisory Committee meetings and often prescribe remotely. Despite better nurse-pharmacist communication through meetings and review reports, the retrospective nature of these reports delays timely AMS actions. Furthermore, participants identified inadequate communication with patients regarding antimicrobial use and resistance as a major challenge in fulfilling their role in AMS. This can lead to poor patient education, unsatisfactory adherence to prescribed regimens, and a lack of awareness about AMR.

#### Community AMS communication system

The absence of a formal, consistent communication strategy in community AMS hampers the effectiveness of AMS initiatives. Participants emphasised the need for an organised communication structure to ensure the sustainability and effectiveness of AMS efforts. Also, some respondents stressed that engaging external stakeholders is essential to improving community AMS. This includes governing bodies disseminating updates on AMS strategies and data related to antimicrobial use and resistance to health professionals, particularly prescribers, to improve prescribing practices. A coordinated communication system involving external stakeholders– such as governing bodies, policymakers, and other healthcare organisations– is crucial to the success of community AMS programmes.

### Prescribing, dispensing and surveillance systems

Inadequate and inefficient systems for prescribing, dispensing, and surveillance pose significant challenges to the successful implementation of AMS in community setting. These system limitations hinder appropriate antimicrobial use, reduce the ability to monitor AMR trends, and impede effective evaluation of AMS interventions. Addressing these issues is essential to optimising AMS outcomes in community settings.

#### Audit and surveillance data and system

Participants identified insufficient tracking and reporting of antimicrobial use and resistance data in the community as a limitation, hindering the ability to monitor prescribing practices and evaluate antimicrobial appropriateness. They emphasised an absence of systems to track these effectively. Most interviewees highlighted that time and funding are barriers to monitoring and reporting these data. In addition, they highlighted the importance of local AMR pattern or data in clinical practice, which is essential for guiding empirical antimicrobial therapy and informing medication management reviews.

#### Prescribing and dispensing strategies

Some challenges related to prescribing, medication reviews, and dispensing practices were also reported. These include doctors’ reluctance to change prescribing habits, patients’ expectations for antimicrobials, and insufficient information to assess prescription appropriateness. Additionally, difficulty contacting prescribers, slow microbiological test turnaround times, and limited laboratory hours contribute to delays. The Pharmaceutical Benefits Scheme (PBS), requiring manufacturer pack sizes, complicates AMS efforts by causing leftover medication and a disconnect between prescribed and used antibiotics. In HMR settings, pharmacists struggle to get older patients to follow up with doctors after recommendations. Similarly, in aged care, infrequent medication reviews (every 6–12 months) often miss prolonged antibiotic use, and reports are underutilised due to poor communication with doctors. Moreover, budget constraints prevent nurses from accessing evidence-based guidelines. Nurses also lack access to pathology results, limiting their ability to review prescriptions. Pharmacists are not embedded in aged care, though stakeholders could consider this for future extended pharmacy practice. Another major concern involves challenges with existing software systems. Participants reported the existence of multiple dispensing software programmes that do not communicate effectively, complicating efforts to capture data on non-PBS or private antimicrobial prescriptions. They advocated for government support in developing integrated software linkages for all data required to enhance AMS programme.

### Resources

There are resource limitations that hinder the effectiveness of AMS in the community setting. These encompass both human and non-human resources, affecting various aspects of the programme. They include insufficient staffing, lack of financial support, restricted access to technology, and gaps in education. Addressing these resource limitations is essential for ensuring the successful adoption and sustainability of community AMS programmes.

#### Resource challenges

These refer to barriers in managing or utilising resources effectively. Participants reported the need for increased awareness and education among health professionals, patients, and their families regarding the AMS programme. In addition, poor uptake of resources was reported. AMS strategies have been used to effectively improve prescribing and use of antimicrobials; however, healthcare providers often fail to use available resources due to lack of awareness, time constraints, or insufficient training, leading to inconsistent practices. Without widespread adoption of AMS strategies, the benefits of improved antimicrobial prescribing are not fully realised.

#### Resource gaps

These highlights the absence or insufficiency of necessary resources. Most of the participants emphasised the shortage of healthcare workers required to effectively implement and sustain AMS programmes. Some participants promoted the need for government support to address this shortage, including funding for employment of healthcare workers and public awareness campaigns. Also, participants highlighted the lack of essential tools, technologies, and informational resources, which hinder efforts to optimise antimicrobial prescribing and use in the community.

**Fig. 1:**
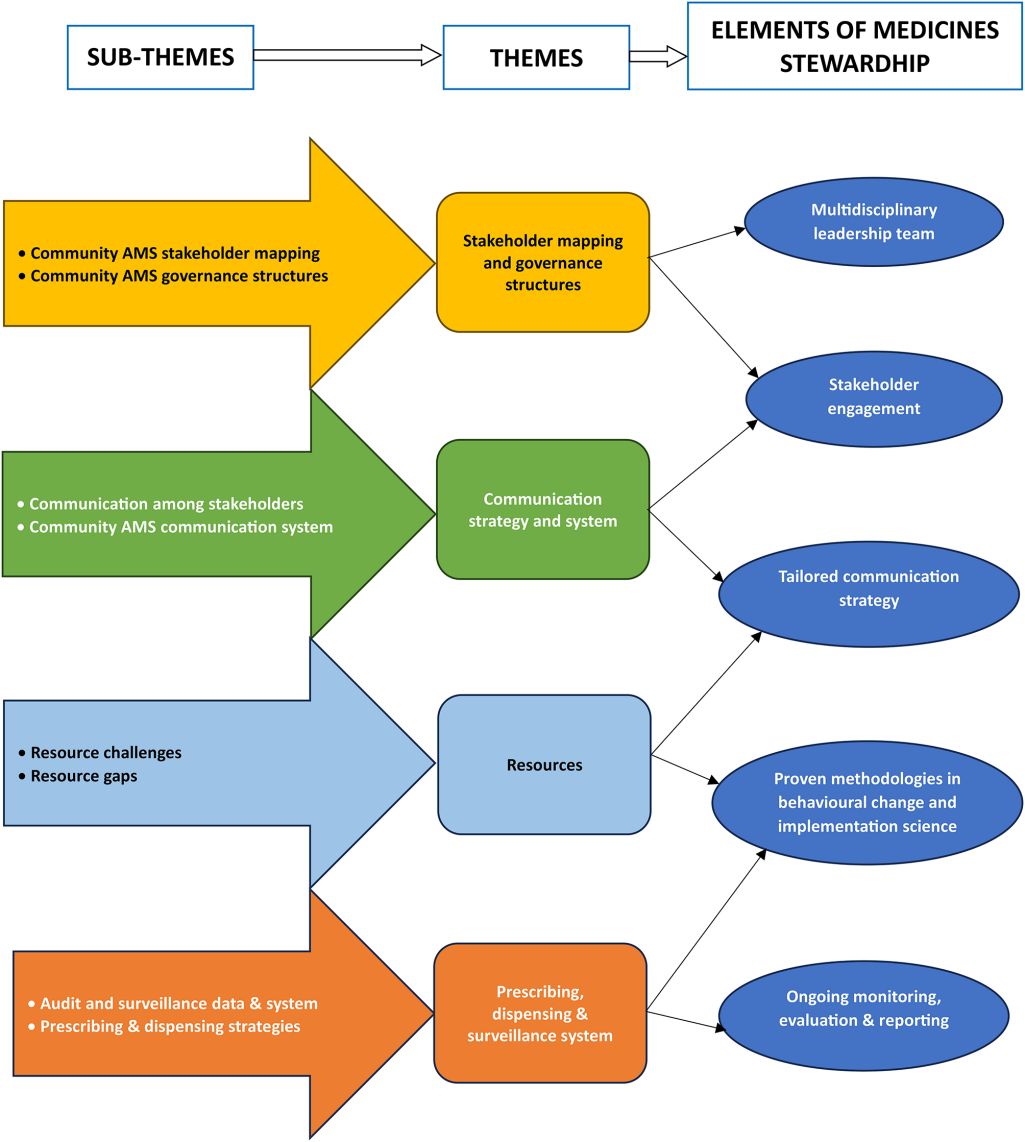
Mapping of themes to relevant elements of medicines stewardship

These findings were mapped and explored using the elements of medicines stewardship (EMS). The mapping of the themes to the respective EMS is represented in Fig. 1.

## Discussion

For successful improvement and implementation of community AMS programmes, it is crucial to examine the current practices, perceptions, and views of health professionals in the community setting regarding AMS practices and systems. To our knowledge, this is the first study utilising qualitative methodology to approach this issue from a varied health professional perspective in diverse practice settings in the community. This study contributes to the body of knowledge on community AMS by providing a deeper understanding and uncovering new insights into the diverse contexts within the community setting, examined through a health system perspective. The interviewees’ responses revealed a lack of clarity and formality in defining the roles and responsibilities of stakeholders involved in community-based AMS initiatives. There is also an absence of comprehensive stakeholder mapping for community AMS, including the absence of external high-level AMS teams and multidisciplinary collaboration. Furthermore, governance structures are insufficient with no clear requirement for AMS in health facilities in the community. Communication among stakeholders, including healthcare providers and patients, is inadequate. Moreover, the absence of a structured communication system and a high-level external communication strategy further undermines AMS efforts. There is a lack of effective data tracking and reporting due to insufficient audit and surveillance infrastructure. Prescribing and dispensing practices face challenges, including inefficiencies in medication reviews and software. Additionally, inadequate awareness and a shortage of both human and non-human resources—such as funding, technology, and training—further hinder the effective implementation of AMS. Consequently, these findings highlight a state of ambiguity and fragmentation in the community AMS practices and systems. In comparison to the hospital-based AMS system, the Australian community AMS system appears to be in its nascent stages of development.

Medicines stewardship programmes aim to optimise the safe and effective use of medicines, tailored to individual needs, while minimising harm to both individuals and society, ultimately enhancing population health outcomes [40]. The elements of medicines stewardship (EMS) provide a structured framework for initiating and enhancing stewardship programmes, with principles that remain consistent across different therapeutic areas and healthcare settings. Operationalising the EMS within this study gives context to our findings, illustrating association with the EMS and relating to the implementation of successful community AMS programmes. Examining our study findings through the lens of the EMS framework underscores the interconnected challenges faced by community AMS initiatives. These findings demonstrate a significant lack of conformity with key elements of effective medicines stewardship [37]. Developing a robust community AMS system using the EMS requires a multidisciplinary leadership team comprising policymakers, medical professionals, nurses, pharmacists, and consumer representatives to ensure coordinated implementation. Incorporating stakeholder mapping and involving key players can facilitate the design and adoption of stewardship models adapted to community needs. Effective communication strategies should harness existing networks to disseminate information and drive behavioural change. In addition, evidence-based approaches from behavioural change strategies and implementation science, such as educational interventions, the appointment of facility-based leads or teams, and the application of modified pilot programmes, are critical to enhancing AMS efforts. Ongoing monitoring and evaluation, using a combination of structural, process, outcome, and balancing measures, are also essential. These measures should include monitoring compliance with prescribing guidelines, evaluating patient outcomes, and tracking adverse events to continuously refine and optimise stewardship strategies. Therefore, based on these approaches, the EMS serves as a valuable framework for developing and implementing community AMS strategies that align with its core principles [37].

Our study highlights the complex and multifaceted challenges faced by community AMS programmes, which is consistent with findings from other studies that identified gaps in knowledge and awareness among healthcare professionals, as well as insufficient resources [41–43]. Factors such as pressure from patients to prescribe antimicrobials, time constraints impacting prescribing decisions, and limited opportunities for AMS-related professional training have also been noted [42, 43]. Furthermore, the absence of a multidisciplinary approach, inadequate governance structures, and a lack of communication and collaboration among healthcare professionals have been emphasised [43]. The results of this study align with findings from an analysis of Australia’s National Action Plan (NAP) on AMR, which identified fragmented implementation and unclear strategic objectives, and recommended strengthening governance, surveillance systems, and stakeholder engagement to enhance AMR strategies [44]. Many governments have developed their NAP but having a NAP does not guarantee that the suggested strategies to address AMR will be implemented or mandated [45–47]. Although the NAP establishes the strategies, the health system is responsible for developing and executing AMS initiatives to achieve goals [48]. A health systems approach is essential for developing an effective AMS system in the community setting [43]. All the health system building blocks—service delivery, health workforce, health information systems, access to medical products, vaccines, and technologies, financing, and leadership and governance—are required to improving AMS within the healthcare system [49, 50]. By aligning AMS with these key components, healthcare systems can optimise antimicrobial use, combat AMR, and improve patient outcomes, ensuring the sustainability of effective antimicrobial treatments. Another study identified key health system components—governance, education, consultation support, healthcare professionals’ involvement, monitoring and feedback, and research—as essential elements for developing a framework to guide AMS in the community [51]. These components closely align with the WHO health system building blocks and the Core Elements of Antibiotic Stewardship outlined by the United States CDC [38, 49]. The implementation and uptake of AMS interventions require governance linked to the framework of the Core Elements of Antibiotic Stewardship, supported by sustainable funding and collaboration with stakeholders [52]. This approach aligns with our proposed application of the EMS in this study. Notably, the EMS aligns closely with the Core Elements of Antibiotic Stewardship. These frameworks underscore the importance of structured systems and processes to effectively guide and sustain AMS initiatives.

In Australia, all health service organisations are required to have an AMS programme; however, its formal establishment and implementation in the community setting remain limited compared to the hospital setting [53–55]. Moreover, the policy frameworks and governance structures to facilitate the development and implementation of community AMS is deficient [51, 54, 56]. The implementation of AMS programmes in the community setting remains a significant challenge, even in high-quality healthcare systems such as those in Australia and other developed countries [43, 57]. The community setting is a critical area for AMS initiatives and should be prioritised for the implementation of AMS programmes, as it accounts for the majority of antimicrobial prescriptions [43, 58, 59]. However, the monitoring and reporting of defined measures or quality indicators within this setting remain insufficient. There is a lack of AMS benchmarking in the community setting, highlighting the need to develop and strengthen strategies, such as antimicrobial prescribing appropriateness using evidence-informed performance indicators [60]. The findings of this study emphasise the critical need for an efficient data tracking and reporting system in the community setting. Granular data on antimicrobial use and resistance is essential for accurately monitoring AMR and predicting future trends. Surveillance efforts tend to focus disproportionately on hospital settings, leading to gaps in understanding resistance trends in community setting. Also, the lack of data integration and interoperability, inconsistencies in prescribing and dispensing practices and absence of granular data hinder the effectiveness of real-time AMS interventions. Additionally, ethical and legal challenges, including privacy concerns, data-sharing limitations, and institutional policies, further complicate the collection and integration of comprehensive surveillance data in the community setting. Addressing these challenges requires optimised system interoperability, standardised reporting protocols, and policies that reconcile data accessibility with ethical considerations, thereby improving the effectiveness of AMS programmes. Qualitative research with health professionals across various settings have enhanced the understanding of contextual determinants concerning AMS programme implementation [57]. To guarantee that stewardship is a standard part of routine care, AMS programmes should be integrated into all relevant frameworks and clinical governance systems with executive oversight and reporting structures (37, 40).

This research has its own strengths and weaknesses. This was an in-depth qualitative study that incorporated the techniques used to ensure rigor, credibility, and trustworthiness in research. This study employed data source triangulation by incorporating input from various health professionals and diverse community practice settings. This approach strengthened data credibility and minimised recruitment bias. Another strength of this research was the use of the EMS within the study which gives context to the themes relating to the implementation of successful community AMS programmes. Also, a standardised framework for reporting qualitative research– COREQ, was used to ensure transparency, thoroughness, and completeness, enabling the assessment of the study’s trustworthiness and replicability. Though data saturation was achieved, the relatively small sample size limits the generalisability of the findings. Time constraints and restricted access to the study population influenced the sample size, and the lack of demographic data further restricts the ability to explore potential variations in perspectives. Future studies should consider broader recruitment strategies and the inclusion of demographic data to capture a wider range of views and provide deeper insights. Another limitation is that data transcription was performed by a single researcher, which may have introduced bias or error, still efforts were made to ensure accuracy. While respondent validation was performed, the validation rate was low. However, the validated transcripts aligned with the interview data, suggesting that the non-validated transcripts likely reflected similar findings. Also, one researcher conducted the data analysis, with deliberation on codes and themes by the research team to minimise bias, though independent verification of the analysis by an external researcher would have further strengthened the study’s objectivity. Although the responses were collected from key informants—health professionals in community settings in South-East Queensland, Australia—the findings may not fully represent the perspectives of health professionals in other Australian states. Furthermore, the findings may not be directly transferable to other countries’ contexts, though they hold relevance for developed nations. Nevertheless, the qualitative data from this study may serve as a baseline for future research. While the researcher’s background in health services research and antimicrobial stewardship may have influenced the research focus, every effort was made to ensure an objective interpretation of the findings. A rigorous approach, including data triangulation and comprehensive analysis, was employed to minimise potential bias and ensure that the conclusions were grounded in the data. Despite these limitations, this study is nonetheless valid for the purpose of answering the research questions.

Future studies utilising the knowledge gained from this study may provide a structure and context for AMS in community settings. Key priorities should include developing a quality improvement plan through research that actively engages policy and consumer stakeholders, utilising co-design or stakeholder involvement methodologies. Future research is needed to develop AMS frameworks adapted to community setting as well as consolidation of EMS into relevant frameworks. The current research identifies areas where activities related to community AMS can be optimised. The Australian Commission on Safety and Quality in Health Care has mandated all acute care providers to implement AMS programmes, supported by expert multidisciplinary teams and robust processes to guide and monitor the appropriate use of antimicrobials within their organisations [54]. This requirement should be extended to all health facilities in the community setting where antimicrobials are prescribed, in order to have more efficient and successful AMS interventions. There are contextual peculiarities within healthcare practices in the community setting, however community-based AMS strategies can be adapted to these specific contexts. Such strategies play a crucial role in reducing AMR by promoting appropriate antimicrobial use and improving prescribing practices within the community. Establishing a health facility-based AMS focal person or team is crucial in community settings, with support from an external multidisciplinary AMS team, potentially operating at the Primary Health Network level. It is important to allocate sufficient time and resources for AMS focal persons or teams to participate in relevant activities and foster complementary skills such as implementation science and information systems.

## Conclusion

This study has provided critical insights into current community AMS practices, highlighting key challenges and opportunities for improvement and serve as a foundation for developing community specific AMS system. The study identified key health system components that influence the effective implementation of community AMS programmes and underscored the need for developing community-specific AMS governance and frameworks. These should integrate multidisciplinary strategies to facilitate implementation and improve patient outcomes.

Given that most antimicrobial usage occurs in the community setting, this study underlines the importance of epidemiology and health service research to support AMS strategies, contributing to broader sustainable development goals. Findings from this research will help inform intervention strategies related to community AMS which have implications for policy and practice to drive sustainable advancements in community healthcare and combat antimicrobial resistance.

## Electronic supplementary material

Below is the link to the electronic supplementary material.

Supplementary Material 1

Supplementary Material 2

Supplementary Material 3

Supplementary Material 4

Supplementary Material 5

## Data Availability

The transcripts generated and data analysed during this study are not yet publicly available because some of the data will be utilised in subsequent publications. Some materials are included in this published article as supplementary while others are not publicly available because they contain sensitive information. However, data and other materials for this study are available from the corresponding authors on request.

## Abbreviations

AMR: Antimicrobial resistance
AMS: Antimicrobial stewardship
CDC: Centers for Disease Control and Prevention
COREQ: Consolidated criteria for reporting qualitative research
EMS: Elements of Medicines Stewardship
NAP: National Action Plan
WHO: World Health Organisation
