# Correction: Antimicrobial stewardship in the community setting: a qualitative exploratory study

**DOI:** 10.1186/s13756-025-01556-z

**Published:** 2025-04-23

**Authors:** Rose I Okonkwo, Henry Ndukwe, Gary Grant, Sohil Khan

**Affiliations:** 1 School of Pharmacy and Medical Sciences, Griffith University, Gold Coast, QLD Australia; 2 Manipal College of Pharmaceutical Sciences and Prasanna School of Public Health, Manipal Academy of Higher Education, Manipal, India

**Antimicrobial Resistance & Infection Control (2025) 14:9**

10.1186/s13756-025-01524-7.

The original article has been updated to amend a typographical error in which Fig. 1 was erroneously cut-off at the margin due to an error carried forward by the production team that handled the article.

