# Supplementary material for: Antimicrobial stewardship in the community setting: a qualitative exploratory study
Source: Antimicrob Resist Infect Control. 2025 Feb 11;14:9. doi: 10.1186/s13756-025-01524-7 (PMC11816747; doi:10.1186/s13756-025-01524-7)
Supplement: Supplementary file 1 — Supplementary Material 1 [file 13756_2025_1524_MOESM1_ESM.pdf]

Griffith UNIVERSITY
Queensland, Australia

GU ref no: 2022/537

Interview Guide for Medical Doctor

Project Title: Development of a quality improvement strategy for antimicrobial stewardship in the community setting

	Theme	Interview Question	Prompts
1	**Role in antimicrobial stewardship (AMS)**	What are your thoughts on AMS (approaches to ensuring/optimising antimicrobial use) in the community setting?	• What is/ are your practice setting(s) in the community? (e.g., GP clinic, outpatient clinic, aged care etc.)<br>• What is your role in optimising antimicrobial use (i.e., AMS) in your practice setting? What has been your role regarding the AMS?<br>• What is the role of doctors in AMS in your community setting'? Are there any other AMS roles you think doctors should carry out in the community setting?<br>• What challenges have you experienced in implementing or carrying out your role in AMS in this setting?
2	**Prescribing practices of antimicrobials in the community setting**	What determines optimal antimicrobial prescribing in your practice in aged care homes and/or general practice?	• What informs the choice of an appropriate empiric antimicrobial therapy in your practice?<br>• What are the factors that may impact antimicrobial prescribing in your practice in aged care homes and/or general practice?<br>○ Resources or clinical experience – any challenges and limitations<br>○ Patient preference – any challenges and limitations<br>○ Logistics of microbiological testing<br>○ Geo-located information on AMR – Knowledge of local antimicrobial resistance patterns is one of the elements that can be used to determine the choice of an appropriate empiric antimicrobial therapy, how would this better inform prescribing?<br>• Are there any formulary restrictions that impact your practice in the community setting?

			• *In Australia, the Antimicrobial Stewardship Clinical Care Standard aims to ensure that a patient with an infection receives optimal treatment - the right antibiotic, at the right dose, by the right route, for the right duration based on accurate assessment and timely review according to the TG and same documented with the indication in the patient's health record*, what are your approaches to ensuring the implementation of Antimicrobial Stewardship Clinical Care Standard in your practice?<br>• What support (resources/ tools) do you require to optimise antimicrobial prescribing and adherence to guidelines in your practice?<br>• At what point do you request microbiological testing for targeted therapy? What informs microbiological testing in your practice? What are the challenges regarding microbiological testing that may impact AMS practice/ optimising antimicrobial prescribing in the community setting?<br>• What procedures/ measures do you take to provide feedback on challenges in your prescribing practice?
**3**	**Use of health organisation's surveillance data for improvement in antimicrobial prescribing and use**	How has your practice surveillance data improved your prescribing practice and compliance with antimicrobial guidelines?<br><br>*Surveillance data i.e., data on healthcare-associated infections (HAIs), antimicrobial use (AMU) and antimicrobial resistance (AMR)	• What are the challenges to the provision of surveillance and data analysis on HAIs, AMU and AMR in your health facility? (if not in practice, then ask;<br>o how do you think facility surveillance data will improve your prescribing practice?<br>o what are the barriers to the provision of surveillance and data analysis on HAIs, AMU and AMR in your health facility?)<br>• What are the areas of action for improvement in appropriateness of antimicrobial prescribing in your practice?<br>• How effective is the existing communication system between you and pharmacist, and the rest of the AMS/ Infection Prevention Control (IPC) team in your practice? Does it exist? Any changes?<br>• What system is available to provide feedback to governing bodies on areas of action to improve antimicrobial prescribing and use/ AMS?
**4**	**AMS program in the community setting**	AMS strategies have been used to effectively change practice behaviour of prescribers since its implementation in Australia, what are your thoughts on this?	• How have these AMS interventions influenced antimicrobial prescribing in your community practice setting?<br>• What are the challenges of implementing AMS strategies/ optimising antimicrobial prescribing and use in the community setting ?<br>• What approaches are used for monitoring and evaluating antimicrobial prescribing and use/ AMS activities in the community setting?

			• How do you think antimicrobial prescribing can be improved in the community setting particularly aged care? How do you think doctors/GPs can improve antimicrobial prescribing and use in the community?<br>• What is the feasibility (possibility/ practicality) of a team of Doctor-Pharmacist or Doctor-Pharmacist-Nurse manage AMS in the comm setting e.g. aged care, general practice or outpatient clinic?
**5**	**COVID-19 pandemic context**	How has the AMS practices in GP and Aged care settings changed post COVID-19 pandemic?	• How has AMS practices and attitudes changed post COVID-19 pandemic?<br>• How do you think the COVID-19 pandemic influenced your prescribing?

We have almost come to the end of our interview; do you have any comments or suggestions to add?

Thank you.
