# Supplementary material for: Antimicrobial stewardship in the community setting: a qualitative exploratory study
Source: Antimicrob Resist Infect Control. 2025 Feb 11;14:9. doi: 10.1186/s13756-025-01524-7 (PMC11816747; doi:10.1186/s13756-025-01524-7)
Supplement: Supplementary file 2 — Supplementary Material 2 [file 13756_2025_1524_MOESM2_ESM.pdf]

Griffith UNIVERSITY
Queensland, Australia

GU ref no: 2022/537

Interview Guide for Medical Laboratory Scientist and/ Manager

Project Title: Development of a quality improvement strategy for antimicrobial stewardship in the community setting

	Theme	Interview Question	Prompts
1	**Role in antimicrobial stewardship (AMS)**	What are your thoughts on AMS (approaches to optimising antimicrobial use) in the community setting?	• How do you think your role as a Medical Laboratory Scientist/ Laboratory Manager contributes to AMS (optimising antimicrobial use) and impact AMS/ AMR strategy in the community setting?<br>• What challenges have you experienced in implementing or carrying out your role in impacting AMS or AMR strategy?<br>• What are the areas of improvement regarding the role of laboratory managers in contributes to AMS (optimising antimicrobial use) and impact AMR strategy in the community setting?
2	**Reporting and use of health services organisation's surveillance data and microbiological tests for improvement in antimicrobial prescribing and use**	How do you think sharing health services organisation's AMR surveillance data with respective organisations will improve antimicrobial prescribing/ AMS practice?<br><br>*Surveillance data i.e., data on healthcare-associated infections (HAIs), antimicrobial use (AMU) and antimicrobial resistance (AMR)	• What are the challenges and barriers to the provision of AMR surveillance data to health services organisations (HSOs)?<br>• How can routine reporting of AMR surveillance data to HSOs be embedded in the community setting?<br>• How do you think geo-located information on AMR will be valuable in guiding antimicrobial prescribing and use in the community?<br>• How effective is the existing communication system between laboratory managers and AMS/AMR policy stakeholders? Does it exist? Any challenges?<br>• What system is available to provide feedback to clinicians and governing bodies on areas to improve AMS practice or AMR strategy?
3	**AMS program in the community setting**	How do you think the microbiological testing practice(s) has impacted AMS strategies/ optimising antimicrobial prescribing in the community setting?	• What are the challenges regarding microbiological testing in your practice that may impact AMS strategies/ optimising antimicrobial prescribing in the community setting?<br>• How does the equipment your lab is furnished with impact turnaround time of microbiological test results? *NB: Some labs have appropriate automation to allow for faster turnaround time

4	**COVID-19 pandemic context**	How has the AMS practices in the community setting changed post COVID-19 pandemic?	• How do you think the COVID-19 pandemic has influenced microbiological testing in the community setting?

We have almost come to the end of our interview; do you have any comments or suggestions to add?

Thank you.
