# Supplementary material for: Antimicrobial stewardship in the community setting: a qualitative exploratory study
Source: Antimicrob Resist Infect Control. 2025 Feb 11;14:9. doi: 10.1186/s13756-025-01524-7 (PMC11816747; doi:10.1186/s13756-025-01524-7)
Supplement: Supplementary file 3 — Supplementary Material 3 [file 13756_2025_1524_MOESM3_ESM.pdf]

GU ref no: 2022/537

Interview Guide for Nurse/ Antimicrobial Steward/ Infection Prevention Practitioner

Project Title: Development of a quality improvement strategy for antimicrobial stewardship in the community setting

	Theme	Interview Question	Prompts
1	**Role in antimicrobial stewardship (AMS)**	What are your thoughts on AMS (approaches to optimising antimicrobial use) in the community setting?	• What is/ are your practice setting(s) in the community? (e.g., general practice clinic, outpatient clinic and/ aged care)<br>• What is your role in AMS in your practice setting? What has been your role regarding the AMS?<br>• What is the role of nurses in AMS in your community setting?<br>• What challenges have you experienced in implementing or carrying out your role in AMS?
2	**Use of health organisation's surveillance data for improvement in antimicrobial prescribing and use**	How has your practice surveillance data improved antimicrobial prescribing and use /AMS practice and compliance with antimicrobial guidelines?<br><br>*Surveillance data i.e., data on healthcare-associated infections (HAIs), antimicrobial use (AMU) and antimicrobial resistance (AMR)	• What are the challenges to the provision of surveillance and data analysis on HAIs, AMU and AMR in your health facility? <u>(if not in practice, then ask;</u><br>○ how do you think facility surveillance data will improve antimicrobial prescribing and use /AMS practice?<br>○ what are the barriers to the provision of surveillance and data analysis on HAIs, AMU and AMR in your health facility?)<br>• What are the areas of action for improvement in appropriateness of antimicrobial use in your practice?<br>• How effective is the existing communication system between you, and medical doctor & the AMS/ Infection Prevention Control (IPC) team in your practice? Does it exist? any changes?<br>• What system is available to provide feedback to clinicians and governing bodies on areas of action to improve antimicrobial prescribing and use/ AMS?
3	**AMS program in the community setting**	How do you ensure the implementation of effective Infection Prevention and	• How is the implementation of effective infection prevention and control (IPC) and AMS strategies carried out in your practice? Is there a person or team responsible for AMS

		Control (IPC) and AMS strategies are carried out in the general practice and aged care setting?	program in your practice? Is there a person or team responsible for IPC program in your practice? If it is conducted continue to next question otherwise move to the last question in this section<br>• What approaches are used for monitoring antimicrobial prescribing and use? How do you evaluate AMS activities in the community setting?<br>• What are the challenges of implementing AMS strategies in the community setting?<br>• What support (resources/ tools) do you require to help optimise antimicrobial use/ AMS in the community setting?<br>• How do you think antimicrobial prescribing and use/ AMS can be improved in (your setting) the community setting particularly aged care? How do you think nurses can improve AMS in the community?<br>• What is the feasibility (possibility/ practicality) of a team of Doctor-Pharmacist or Doctor-Pharmacist-Nurse manage AMS in the comm setting e.g. aged care, general practice or outpatient clinic?
**4**	**COVID-19 pandemic context**	How has the AMS practices in the community setting changed post COVID-19 pandemic?	• How has AMS practices and attitudes changed post COVID-19 pandemic?<br>• How do you think the COVID-19 pandemic has influenced antimicrobial prescribing and use?<br>• What were the best practices or interventions during the COVID-19 pandemic that improved AMU in the community setting in 2020?<br>• How can these practices be effectively implemented post COVID-19 community lockdown - to improve and sustain a lower volume of AMU?

We have almost come to the end of our interview; do you have any comments or suggestions to add?

Thank you.
