# Supplementary material for: Antimicrobial stewardship in the community setting: a qualitative exploratory study
Source: Antimicrob Resist Infect Control. 2025 Feb 11;14:9. doi: 10.1186/s13756-025-01524-7 (PMC11816747; doi:10.1186/s13756-025-01524-7)
Supplement: Supplementary file 4 — Supplementary Material 4 [file 13756_2025_1524_MOESM4_ESM.pdf]

GU ref no: 2022/537

Interview Guide for Pharmacist

Project Title: Development of a quality improvement strategy for antimicrobial stewardship in the community setting

	Theme	Interview Question	Prompts
1	**Role in antimicrobial stewardship (AMS)**	What are your thoughts on AMS (approaches to ensuring/optimising antimicrobial use) in the community setting?	• What is/ are your practice setting(s) in the community? (e.g., general practice clinic, outpatient clinic, aged care and/ community pharmacies, Home Medication Review (HMR) etc.)<br>• What is your role in AMS in your practice setting? What has been your role regarding the AMS? In other word, has there been changes in your role regarding AMS in the past years?<br>• What is the role of pharmacists in AMS in your community setting'?<br>• What challenges have you experienced in implementing or carrying out your role in AMS in this setting?
2	**Prescribing practices of antimicrobials in the community setting**	What determines optimal antimicrobial use in the community practice setting?	<u>NB: This first question is not relevant for pharmacists practicing only in community pharmacy.</u><br>• What are the factors that may impact medication management reviews (MMRs) and Quality Use of Medicines (QUM) services in your practice setting? – Chronic diseases e.g. diabetic foot ulcer. Acute infections e.g., UTI, skin & soft tissues infections.<br>○ Resources or clinical experience – any challenges and limitations<br>○ Patient preference – any challenges and limitations<br>○ Logistics of microbiological testing<br>○ Geo-located information on AMR – Knowledge of local antimicrobial resistance patterns is one of the elements that can be used to determine the choice of an appropriate empiric antimicrobial therapy, how would this better inform your MMR and QUM services in your practice?

			• What support (resources/ tools) do you require to optimise antimicrobial use and adherence to guidelines in your practice? (For community pharmacists, replace the underlined words with "to optimise AMS services in your practice?")<br>• *In Australia, the Antimicrobial Stewardship Clinical Care Standard aims to ensure that a patient with an infection receives optimal treatment - the right antibiotic, at the right dose, by the right route, for the right duration based on accurate assessment and timely review according to the TG and same documented with the indication in the patient's health record,* what are your approaches to ensuring the implementation of Antimicrobial Stewardship Clinical Care Standard in your practice?<br>• What procedures/ measures do you take to provide feedback on challenges in your prescribing practice? (NB: For pharmacists involved in prescribing)
**3**	**Use of health organisation's surveillance data for improvement in antimicrobial prescribing and use**	How has your practice surveillance data improved your MMR/ QUM/AMS practice and compliance with antimicrobial guidelines?<br><br>*Surveillance data i.e., data on healthcare-associated infections (HAIs), antimicrobial use (AMU) and antimicrobial resistance (AMR)	• What are the challenges to the provision of surveillance and data analysis on HAIs, AMU and AMR in your health facility? (if not in practice, ask;<br>o how do you think facility surveillance data will improve your MMR/ QUM/AMS practice?<br>o what are the barriers to the provision of surveillance and data analysis on HAIs, AMU and AMR in your health facility?)<br>• What are the areas of action for improvement in appropriateness of antimicrobial use in your practice?<br>• How effective is the existing communication system between you, and General Practitioners & the rest of the AMS/ Infection Prevention Control (IPC) team in your practice? Does it exist? any changes? (Exclude the underlined words for interviews with community pharmacists)<br>• What system is available to provide feedback to clinicians and governing bodies on areas of action to improve antimicrobial prescribing and use/ AMS?
**4**	**AMS program in the community setting**	How do you ensure the implementation of effective AMS strategies are carried out in your community practice setting?	• What approaches are used for monitoring and evaluating antimicrobial prescribing and use/ AMS activities in the community setting?<br>• What are the challenges of implementing AMS strategies/ optimising antimicrobial prescribing and use in the community setting?<br>• How do you think antimicrobial use/ AMS practices can be improved in the community setting particularly aged care? How do you think pharmacists can improve AMS in the community?<br>• What is the feasibility (possibility/ practicality) of a team of D/P and N manage AMS in the comm setting e.g aged care, general practice, outpatient clinic? (collaborative team)

5	**Non-PBS prescribing or dispensing of antimicrobials.**	How can the data from non-PBS prescribing, or private dispensing of antimicrobials be captured?<br><br>*The Pharmaceutical Benefits Scheme (PBS) subsidises medications if they are prescribed for an approved indication. Prescribers can write a private prescription for unapproved indications, but patients will pay the full cost of the medication.<br><br>There are limited reporting mechanisms available for non-PBS/RPBS or private prescriptions. This is an important gap in the surveillance of antimicrobial use and appropriateness in Australia.	*Evidence from AURA,*<br>*Restrictions on PBS-listed antimicrobials may be increasing private prescription.*<br>*In addition, the commercial initiative that enables patient access to online prescribers in community pharmacy settings for a range of medications, including antibiotics may be increasing private prescription.*<br>*These arrangements are outside the scope of the Medical Benefits Schedule telehealth items. The PBS and RPBS do not capture data on private prescriptions.*<br>• How is the volume of antimicrobials dispensed on private prescriptions currently monitored?<br>• How do you think this data on private prescriptions can be monitored and made available to provide a complete picture of AMU in Australia?
6	**COVID-19 pandemic context**	How has the AMS practices in the community setting changed post COVID-19 pandemic?	• How has AMS practices and attitudes changed post COVID-19 pandemic?<br>• How do you think the COVID-19 pandemic has influenced antimicrobial use?

We have almost come to the end of our interview; do you have any comments or suggestions to add?

Thank you.
