# Supplementary material for: Antimicrobial stewardship in the community setting: a qualitative exploratory study
Source: Antimicrob Resist Infect Control. 2025 Feb 11;14:9. doi: 10.1186/s13756-025-01524-7 (PMC11816747; doi:10.1186/s13756-025-01524-7)
Supplement: Supplementary file 5 — Supplementary Material 5 [file 13756_2025_1524_MOESM5_ESM.docx]

**Table 1: Consolidated criteria for reporting qualitative studies (COREQ): 32-item checklist**

| **No** | **Item** | **Guide questions/description** | **Reported in section and notes** |
| --- | --- | --- | --- |
| **Domain 1: Research team and reflexivity** | | | |
| ***Personal Characteristics*** | | | |
| 1 | Interviewer/facilitator | Which author/s conducted the interview or focus group? | All interviews were conducted by R.I.O. This is reported in the methods section. |
| 2 | Credentials | What were the researcher’s credentials? E.g. PhD, MD | R.I.O. (MSc.), H.N. (PhD), G.G. (PhD) and S.K. (PhD) |
| 3 | Occupation | What was their occupation at the time of the study? | R.I.O. (PhD Student), H.N. (Lecturer), G.G. (Associate Professor) and S.K. (Associate Professor) |
| 4 | Gender | Was the researcher male or female? | R.I.O. (Female), H.N. (Male), G.G. (Male) and S.K. (Male) |
| 5 | Experience and training | What experience or training did the researcher have? | Researchers have experience and training in qualitative research methods either as principal investigators or researchers. |
| ***Relationship with participants*** | | | |
| 6 | Relationship established | Was a relationship established prior to study commencement? | The researcher did not have any relationship with participants prior to obtaining expression of interest and consent from participants. |
| 7 | Participant knowledge of the interviewer | What did the participants know about the researcher? e.g. personal goals, reasons for doing the research | Participants were aware that this was a PhD research project and were informed about the goals of the study. All participants gave consent to be included in this study. This is reported in the methods section. |
| 8 | Interviewer characteristics | What characteristics were reported about the interviewer/facilitator? e.g. Bias, assumptions, reasons and interests in the research topic | The research team had an interest in current AMS practices and systems, the challenges, areas for improvement and strategies to optimise AMS. This is reported in all the sections. |
| **Domain 2: Study design** | | | |
| ***Theoretical framework*** | | | |
| 9 | Methodological orientation and Theory | What methodological orientation was stated to underpin the study? e.g. grounded theory, discourse analysis, ethnography, phenomenology, content analysis | We undertook a qualitative study using thematic  analysis for analysing the qualitative interview data. This is reported in the methods section. |
| ***Participant selection*** | | | |
| 10 | Sampling | How were participants selected? e.g. purposive, convenience, consecutive, snowball | This is reported in the methods section. |
| 11 | Method of approach | How were participants approached? e.g. face-to-face, telephone, mail, email | This is reported in the methods section. |
| 12 | Sample size | How many participants were in the study? | This is reported in the methods section. |
| 13 | Non-participation | How many people refused to participate or dropped out? Reasons? | This is reported in the methods section. |
| ***Setting*** | | | |
| 14 | Setting of data collection | Where was the data collected? e.g. home, clinic, workplace | This is reported in the methods section. |
| 15 | Presence of non-participants | Was anyone else present besides the participants and researchers? | This was a one-on-one interview. This is reported in the methods section. |
| 16 | Description of sample | What are the important characteristics of the sample? e.g. demographic data, date | This is reported in the methods section. |
| ***Data collection*** | | | |
| 17 | Interview guide | Were questions, prompts, guides provided by the authors? Was it pilot tested? | This is reported in the methods section. |
| 18 | Repeat interviews | Were repeat interviews carried out? If yes, how many? | There were no repeat interviews. |
| 19 | Audio/visual recording | Did the research use audio or visual recording to collect the data? | This is reported in the methods section. |
| 20 | Field notes | Were field notes made during and/or after the interview or focus group? | Memoing was done. This is reported in the methods section. |
| 21 | Duration | What was the duration of the interviews or focus group? | This is reported in the methods section. |
| 22 | Data saturation | Was data saturation discussed? | This is reported in the methods section. |
| 23 | Transcripts returned | Were transcripts returned to participants for comment and/or correction? | Yes. This is reported in the methods section. |
| **Domain 3: analysis and findings** | | | |
| ***Data analysis*** | | | |
| 24 | Number of data coders | How many data coders coded the data? | One. This is reported in the methods and discussion (study limitations) section. |
| 25 | Description of the coding tree | Did authors provide a description of the coding tree? | Yes. Figure 1 is a representation of thematic map or coding tree. This is reported in the results section. |
| 26 | Derivation of themes | Were themes identified in advance or derived from the data? | Themes were derived from data. This is reported in the methods and results section. |
| 27 | Software? | What software, if applicable, was used to manage the data? | This is reported in the methods section. |
| 28 | Participant checking | Did participants provide feedback on the findings? | Yes. This is reported in the methods section. |
| ***Reporting*** | | | |
| 29 | Quotations presented | Were participant quotations presented to illustrate the themes / findings? Was each quotation identified? e.g. participant number | Quotations are presented in the results section and each quotation was indexed. |
| 30 | Data and findings consistent | Was there consistency between the data presented and the findings? | All findings were derived from the data and all  themes are supported by illustrative quotes. |
| 31 | Clarity of major themes | Were major themes clearly presented in the findings? | Major themes were derived from the data and are clearly defined in sub-sections in the results section. |
| 32 | Clarity of minor themes | Is there a description of diverse cases or discussion of minor themes? | Analysis includes a description of diverse cases and minor themes. Sub-themes were also described. |
